# Holistic Analysis of Coronavirus Literature: A Scientometric Study of the Global Publications Relevant to SARS-CoV-2 (COVID-19), MERS-CoV (MERS) and SARS-CoV (SARS)

**DOI:** 10.1017/dmp.2020.300

**Published:** 2020-08-13

**Authors:** Engin Şenel, Fatih Esad Topal

**Affiliations:** Hitit University Faculty of Medicine, Department of Dermatology and Venereology, Çorum, Turkey; Hitit University, Traditional and Complementary Medicine Application and Research Center, Çorum, Turkey; Katip Çelebi University Ataturk Training and Research Hospital, Department of Emergency, Izmir, Turkey

**Keywords:** bibliometrics, coronavirus, COVID-19, MERS, SARS, SARS-CoV-2, SARS-CoV, scientometrics

## Abstract

**Objectives:**

In late December 2019, a cluster of patients with pneumonia caused by an unknown pathogen was reported from Wuhan, Hubei Province, China. The pathogen has been identified as a novel coronavirus, severe acute respiratory syndrome 2 (SARS-CoV-2) and the disease has been named as coronavirus disease 2019 (COVID-19). The objective of this study was to perform the first holistic scientometric evaluation of coronavirus publications.

**Methods:**

Our main source for this study was Web of Science Collection database. All items published between 1980 and 2019 were included. A distribution map of global production in coronavirus literature and scientometric networks were generated.

**Results:**

The United States, China, Germany, the United Kingdom, and Netherlands were the most productive countries. Publications in coronavirus literature have been produced from almost every country in the world, except for some countries in Asia and Africa.

**Conclusion:**

While in the 1980s, the United States and developed countries from Europe were major source countries and the virus was identified only as an animal disease in the literature and its biological and genetic structure was investigated, in the 2000s, China became a major contributor of coronavirus literature because the SARS outbreak originated from southern China. Almost all most-cited publications in this period are related to SARS and the ACE2 protein.

In late December 2019, a cluster of cases with pneumonia caused by an unknown etiology was reported from Wuhan, Hubei Province, China.^1^ Later, the cause of the disease was identified as a novel betacoronavirus, the 2019 novel coronavirus (2019-nCoV). The disease was coined as coronavirus disease 2019 (COVID-19). Recently, the official name of the virus that causes COVID-19 has been announced by World Health Organization (WHO) as severe acute respiratory syndrome coronavirus 2 (SARS-CoV-2).^2^

Scientometrics, also known as “Science of science,” is a popular statistical method analyzing scientific literature thoroughly in a certain field.^3^ The academic literature lacks an in-depth scientometric study evaluating coronaviruses and disease caused by them. Herein, we aimed to perform a holistic scientometric assessment of the coronavirus literature.

## METHODS

Our main source of this study was Web of Science (WoS, Thomson Reuters, New York, NY) Core Collection database. We preferred WoS as our main data source because it provides data analysis for publications and citations and allows the results to be sorted according to the number of citations. In addition, WoS attribution data are considered more reproducible and reliable than other databases, and WoS is used as the standard by certain official organizations.^4^ We used keywords of “*coronavirus,*” “*SARS*”, “*SARS-CoV*”, “*MERS*”, “*MERS-CoV*”, and “*COVID-19*” for our analysis. All items published between 1980 and 2019 were included, and documents produced in 2020 were excluded for our major analysis. A distribution map of global production of coronavirus literature was generated by a free Web source named GunnMap.^5^ VOSviewer freeware was used to create scientometric networks.^6^

## RESULTS

### General Features of Coronavirus Literature

A total of 13,833 documents indexed in WoS Core Collection between 1980 and 2019 were found in coronavirus literature, 7339 of which were open access. The peak year for publication was 2016 with 837 papers and 106 articles have been produced in 2020 so far. English was the major language of coronavirus literature (96.957%) followed by French, German, Spanish, and Chinese (0.802, 0.766, 0.289, and 0.231, respectively). Original articles covered 80.518% with 11,138 documents of all coronavirus literature followed by reviews, proceeding papers and editorials (8.545, 4.634, and 2.704%, respectively; Table 1). Virology, Veterinary Sciences, and Infectious Diseases were the most studied areas in literature (Table 1).

**TABLE 1:** Features of Coronavirus Literature^a^

	Number	%^b^
**Document Types**		
Original article	11,138	80.518
Review	1,182	8.545
Proceedings paper	641	4.634
Editorial material	374	2.704
Meeting abstract	344	2.487
Book chapter	291	2.104
Letter	256	1.851
Note	185	1.337
News item	82	0.593
Correction	55	0.398
Early access	34	0.246
Book	6	0.043
Correction	3	0.022
Data paper	1	0.007
**Research Areas**		
Virology	4411	31.888
Veterinary sciences	2093	15.130
Infectious diseases	1613	11.661
Immunology	1558	11.263
Microbiology	1519	10.981
Biochemistry	1347	9.738
Biotechnology	816	5.899
Experimental	658	4.757
Multidisciplinary sciences	598	4.323
Internal medicine	459	3.318
**Top 10 Authors**		
Yuen KY (The University of Hong Kong, China)	218	1.576
Perlman S (University of Iowa, USA)	189	1.366
Enjuanes L (Autonomous University of Madrid, Spain)	176	1.272
Baric RS (University of North Carolina, USA)	171	1.236
Weiss SR (University of Pennsylvania, USA)	150	1.084
Drosten C (Berlin Institute of Health, Germany)	148	1.070
Rottier PJM (Utrecht University, The Netherlands)	134	0.969
Woo PCY (The University of Hong Kong, China)	129	0.933
Chan KH (The University of Hong Kong, China)	122	0.882
Lau SKP (The University of Hong Kong, China)	120	0.867
**Top 10 Institutions**		
University of Hong Kong (China)	534	3.860
Chinese Academy of Sciences (China)	396	2.863
Utrecht University (The Netherlands)	335	2.422
University of California System (USA)	333	2.407
National Institutes of Health (USA)	328	2.371
University Of North Carolina (USA)	273	1.974
Centers for Disease Control Prevention (USA)	258	1.865
Chinese University of Hong Kong (China)	229	1.655
University Of North Carolina (USA)	223	1.612
University of Pennsylvania (USA)	216	1.561
**Top 10 Source Titles**		
Journal of Virology	1166	8.429
Virology	510	3.687
Advances in Experimental Medicine and Biology	370	2.675
Journal of General Virology	330	2.386
Archives of Virology	263	1.901
Emerging Infectious Diseases	245	1.771
Virus Research	245	1.771
PLOS One	239	1.728
Veterinary Microbiology	198	1.431
Journal of Virological Methods	171	1.236
***Total***	***13,833***	***100***

Top Cited Manuscripts				
Article	Author	Journal Name/Published in	Total Citation	Average Citations per Year
***1980-1989 (n = 641)***				
Characterization of An Efficient Coronavirus Ribosomal Frameshifting Signal - Requirement for An RNA Pseudoknot	Brierley, I; Digard, P; Inglis, SC	Cell	466	14.56
Coronaviruses - Structure and Genome Expression	Spaan, W; Cavanagh, D; Horzinek, MC	Journal of General Virology	393	11.91
The Biology and Pathogenesis of Coronaviruses	Wege, H; Siddell, S; Termeulen, V	Current Topics in Microbiology and Immunology	303	7.77
Completion of The Sequence of The Genome of The Coronavirus Avian Infectious-Bronchitis Virus	Boursnell, MEG; Brown, TDK; Foulds, IJ; *et al.*	Journal of General Virology	302	8.88
Pathogenicity of Antigenic Variants of Murine Coronavirus JHM Selected with Monoclonal-Antibodies	Fleming, JO; Trousdale, MD; Elzaatari, FAK; *et al.*	Journal of Virology	266	7.60
Isolation of Coronavirus Envelope Glycoproteins and Interaction with The Viral Nucleocapsid	Sturman, LS; Holmes, KV; Behnke, J	Journal of Virology	239	5.83
An Efficient Ribosomal Frame-Shifting Signal in The Polymerase-Encoding Region of The Coronavirus IBV	Brierley, I; Boursnell, MEG; Binns, MM; *et al.*	EMBO Journal	236	6.94
The Biology of Coronaviruses	Siddell, S; Wege, H; Termeulen, V	Journal of General Virology	227	5.97
Coronavirus Genome - Prediction of Putative Functional Domains in The Non-Structural Polyprotein by Comparative Amino-Acid Sequence-Analysis	Gorbalenya, AE; Koonin, EV; Donchenko, AP; *et al.*	Nucleic Acids Research	223	6.97
Proteolytic Cleavage of The E2-Glycoprotein of Murine Coronavirus - Activation of Cell-Fusing Activity of Virions by Trypsin and Separation Of 2 Different 90k-Cleavage Fragments	Sturman, LS; Ricard, CS; Holmes, KV	Journal of Virology	215	5.97
***1990-1999 (n = 1674)***				
Community Study of Role of Viral-Infections in Exacerbations of Asthma In 9-11-Year-Old Children	Johnston, SL; Pattemore, PK; Sanderson, G; *et al.*	British Medical Journal	1328	51.08
Psychological Stress and Susceptibility to The Common Cold	Cohen, S; Tyrrell, DAJ; Smith, AP	New England Journal of Medicine	1005	33.50
Respiratory Viruses and Exacerbations of Asthma in Adults	Nicholson, KG; Kent, J; Ireland, DC	British Medical Journal	796	28.43
Lelystad Virus, The Causative Agent of Porcine Epidemic Abortion and Respiratory Syndrome (Pears), Is Related to LDV and EAV	Meulenberg, JJM; Hulst, MM; Demeijer, EJ; *et al.*	Virology	607	21.68
The Molecular Biology of Coronaviruses	Lai, MMC; Cavanagh, D	Advances in Virus Research	545	22.71
Isolation and Direct Characterization of Resident Microglial Cells from The Normal and Inflamed Central-Nervous-System	Sedgwick, JD; Schwender, S; Imrich, H; *et al.*	Proceedings of The National Academy of Sciences of The United States of America	533	17.77
RNA Recombination in Animal and Plant-Viruses	Lai, MMC	Microbiological Reviews	495	17.07
Porcine Reproductive and Respiratory Syndrome Virus Comparison: Divergent Evolution on Two Continents	Nelsen, CJ; Murtaugh, MP; Faaberg, KS	Journal of Virology	484	22.00
Human Aminopeptidase-N Is A Receptor for Human Coronavirus-229e	Yeager, CL; Ashmun, RA; Williams, RK; *et al.*	Nature	454	15.66
Aminopeptidase-N Is A Major Receptor for The Enteropathogenic Coronavirus TGEV	Delmas, B; Gelfi, J; Lharidon, R; et al.	Nature	425	14.66
***2000-2009 (n = 4180)***				
A novel coronavirus associated with severe acute respiratory syndrome	Ksiazek, TG; Erdman, D; Goldsmith, CS; *et al.*	New England Journal of Medicine	1829	101.61
Identification of a novel coronavirus in patients with severe acute respiratory syndrome	Drosten, C; Gunther, S; Preiser, W; *et al.*	New England Journal of Medicine	1739	96.61
Characterization of a novel coronavirus associated with severe acute respiratory syndrome	Rota, PA; Oberste, MS; Monroe, SS; *et al.*	Science	1488	82.67
Coronavirus as a possible cause of severe acute respiratory syndrome	Peiris, JSM; Lai, ST; Poon, LLM; *et al.*	Lancet	1439	79.94
The genome sequence of the SARS-associated coronavirus	Marra, MA; Jones, SJM; Astell, CR; *et al.*	Science	1274	70.78
Cloning of a human parvovirus by molecular screening of respiratory tract samples	Allander, T; Tammi, MT; Eriksson, M; *et al.*	Proceedings of The National Academy of Sciences of The United States of America	1021	63.81
Angiotensin-converting enzyme 2 is a functional receptor for the SARS coronavirus	Li, WH; Moore, MJ; Vasilieva, N; *et al.*	Nature	968	53.78
Isolation and characterization of viruses related to the SARS coronavirus from animals in Southern China	Guan, Y; Zheng, BJ; He, YQ; *et al.*	Science	884	49.11
Bats are natural reservoirs of SARS-like coronaviruses	Li, WD; Shi, ZL; Yu, M; *et al.*	Science	844	52.75
Clinical progression and viral load in a community outbreak of coronavirus-associated SARS pneumonia: a prospective study	Peiris, JSM; Chu, CM; Cheng, VCC; *et al.*	Lancet	826	45.89
***2010-2019 (n = 6601)***				
Isolation of a Novel Coronavirus from a Man with Pneumonia in Saudi Arabia	Zaki, Ali Moh; van Boheemen, Sander; Bestebroer, Theo M.; *et al.*	New England Journal of Medicine	1295	143.89
Hospital Outbreak of Middle East Respiratory Syndrome Coronavirus	Assiri, Abdullah; McGeer, Allison; Perl, Trish M.; *et al.*	New England Journal of Medicine	544	68.00
Human infections with the emerging avian influenza A H7N9 virus from wet market poultry: clinical analysis and characterization of viral genome	Chen, Yu; Liang, Weifeng; Yang, Shigui; *et al.*	Lancet	544	68.00
Dipeptidyl peptidase 4 is a functional receptor for the emerging human coronavirus-EMC	Raj, V. Stalin; Mou, Huihui; Smits, Saskia L.; *et al.*	Nature	467	58.38
Viral pneumonia	Ruuskanen, Olli; Lahti, Elina; Jennings, Lance C.; *et al.*	Lancet	448	44.80
Impacts of Bioinformatics to Medicinal Chemistry	Chou, Kuo-Chen	Medicinal Chemistry	379	63.17
Genomic Characterization of a Newly Discovered Coronavirus Associated with Acute Respiratory Distress Syndrome in Humans	van Boheemen, Sander; de Graaf, Miranda; Lauber, Chris; *et al.*	mBio	362	40.22
Epidemiological, demographic, and clinical characteristics of 47 cases of Middle East respiratory syndrome coronavirus disease from Saudi Arabia: a descriptive study	Assiri, Abdullah; Al-Tawfiq, Jaffar A.; Al-Rabeeah, Abdullah A.; *et al.*	Lancet Infectious Diseases	360	45.00
Middle East respiratory syndrome coronavirus neutralising serum antibodies in dromedary camels: a comparative serological study	Reusken, Chantal B. E. M.; Haagmans, Bart L.; Mueller, Marcel A.; *et al.*	Lancet Infectious Diseases	350	43.75
Middle East Respiratory Syndrome Coronavirus (MERS-CoV): Announcement of the Coronavirus Study Group	de Groot, Raoul J.; Baker, Susan C.; Baric, Ralph S.; *et al.*	Journal of Virology	330	41.25

	Top Used Keywords (Occurrences, total link strength)		
	1990–1999	2000–2009	2010–2019
**1**	Coronavirus (64, 125)	Coronavirus (430, 934)	Coronavirus (765, 1176)
**2**	Mouse hepatitis virus (48, 93)	Severe Acute Respiratory Syndrome, SARS, SARS-CoV (314, 584)	Middle East Respiratory Syndrome, MERS-CoV (367, 577)
**3**	Transmissible gastroenteritis virus, TGEV (12, 39)	Spike protein (67, 159)	Epidemiology (136, 238)
**4**	Rotavirus (11, 27)	Nucleocapsid protein (56, 131)	Severe Acute Respiratory Syndrome, SARS, SARS-CoV (122, 172)
**5**	Cat (9, 30)	Infectious bronchitis virus (59, 117)	Porcine epidemic diarrhea virus (112, 131)
**6**	Feline infectious peritonitis (9, 24)	Vaccine (43, 106)	Infectious bronchitis virus (104, 106)
**7**	RT-PCR (9, 14)	Epidemiology (41, 92)	Vaccine (92, 187)
**8**	MHV (9, 12)	RT-PCR (42, 86)	Spike protein (90, 188)
**9**	Demyelination (8, 18)	Rhinovirus (32, 85)	Phylogenetic analysis (85, 110)
**10**	Infectious bronchitis virus (8, 12)	Diagnosis (32, 79)	Respiratory virus(es) (83, 107)
**11**	Porcine (7, 22)	Respiratory syncytial virus (25, 76)	Saudi Arabia (74, 156)
**12**	Gastroenteritis (7, 20)	ACE2 (29, 72)	Influenza (69, 127)
**13**	Feline coronavirus (7, 14)	Influenza (25, 71)	Feline coronavirus (68, 68)
**14**	Porcine respiratory virus (6, 20)	Children (29, 69)	Diagnosis (60, 102)
**15**	Virus (6, 17)	Monoclonal antibody (23, 63)	Respiratory syncytial virus (58, 114)
**16**	Diagnosis (6, 15)	IBV (24, 62)	Feline infectious peritonitis (57, 61)
**17**	Diarrhea (6, 12)	Recombination (20, 55)	Pneumonia (56, 97)
**18**	Pig (6, 11)	Diarrhea (29, 54)	Children (55, 88)
**19**	Serology (5, 14)	Bovine coronavirus (27, 52)	Bat(s) (53, 84)
**20**	Macrophage (5, 13)	Rotavirus (19, 52)	Recombination (49, 91)

a Total percentage may exceed 100% because certain items were included in more than 1 category.

b Of total documents published in coronavirus disease literature.

### Performances of the Countries, Authors, Institutions, and Sources

**FIGURE 1:**
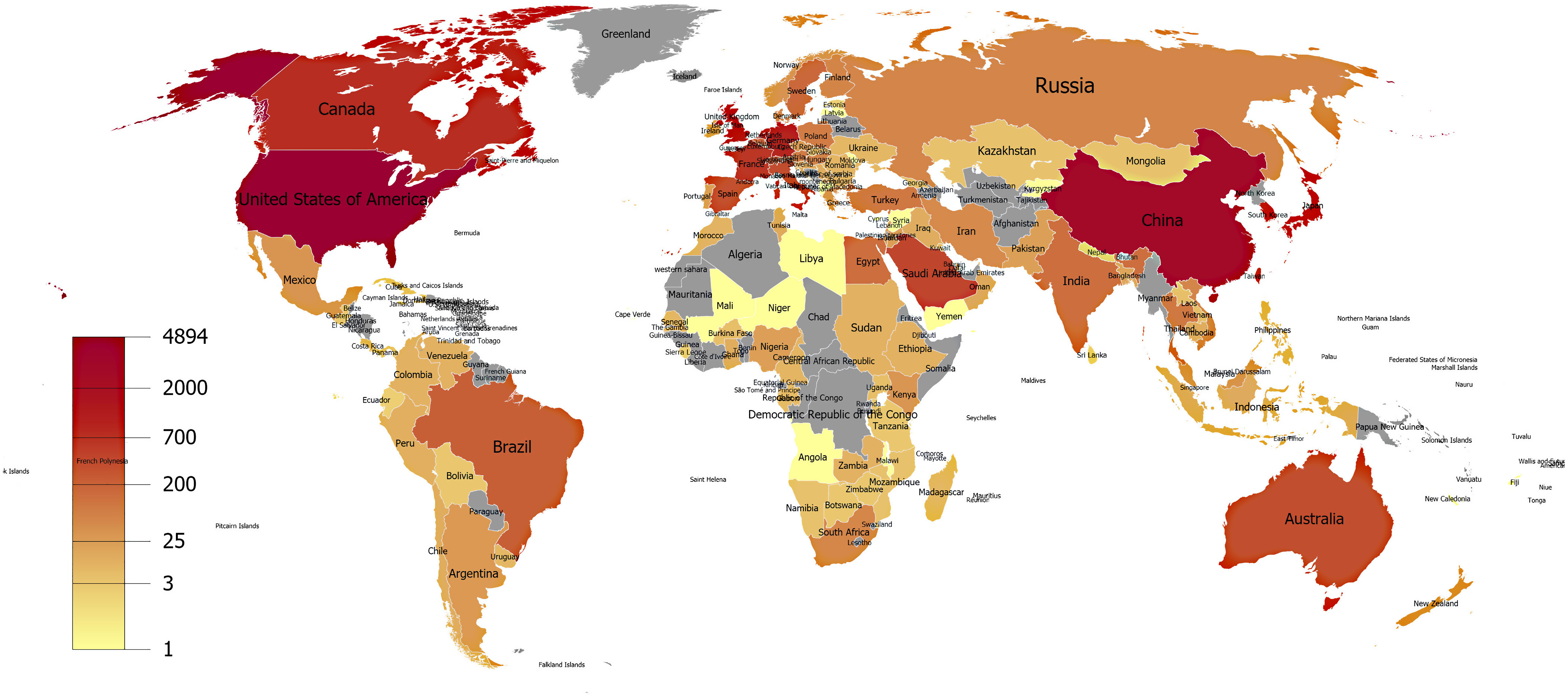
Publication Density of World Countries in Coronavirus Literature.

The United States of America (USA) ranked first in coronavirus literature with 4894 articles (35.379%) followed by China, Germany, the United Kingdom (UK) and the Netherlands (16.663, 6.701, 6.448, and 5.711%, respectively). Publications in coronavirus literature have been produced from almost every country in the world, except for some countries in Asia and Africa (Figure 1). Yuen from the University of Hong Kong (China) was the most prolific author with 218 indexed papers (1.576%, Table 1). University of Hong Kong was also detected as the most productive institution in the literature with 534 publications (3.86%; Table 1). *Journal of Virology*, *Virology* and *Advances in Experimental Medicine and Biology* were the most contributor source titles in coronavirus literature (*N* = 1166, 510, and 370 items, respectively; Table 1).

### Citations, Decades, and Scientometric Networks Analyses

#### 1980-1989

Our search yielded 641 articles in the period of 1980 to 1989. The United States, Federal Republic of Germany, the United Kingdom, Canada, and France dominated coronavirus literature in this period (36.661, 13.417, 12.168, 9.204 and 7.8, respectively). The most prolific author was Termeulen V with 37 items. The *Journal of General Virology* and *University of Würzburg* (Germany) stood out in their categories in this period. H-index of coronavirus literature for this period was calculated as 79 and total number of citations was 21,682 (18,643 without self-citations). Average citations per item were 33.83. The most cited document in this period was an original article titled “*Characterization of An Efficient Coronavirus Ribosomal Frameshifting Signal - Requirement for An RNA Pseudoknot*” written by Brierley et al. (Table 1).

#### 1990-1999

Coronavirus literature had 1674 documents indexed between 1990 and 1999. The United States, Germany, Canada, the United Kingdom, and the Netherlands were the most productive countries between 1990 and 1999 (44.265, 8.961, 8.303, 8.244, and 7.826, respectively). Lai from University of Southern California (USA) was the most prolific author with 70 documents. The *Journal of Virology* was the prominent source title in this decade with 226 articles (13.501%), and *University of Southern California* (USA) was the most contributor institution with 96 papers (5.735%). H-index was measured as 105 and published documents were cited 63,445 times in this period (53,214 without self-citations). The most cited document was an original article titled “*Community Study of Role of Viral-Infections in Exacerbations of Asthma In 9-11-Year-Old Children*” by Johnston et al. (Table 1). As we analyzed scientometric network analysis of coronavirus literature in this period the most indexed keywords were detected to be “*coronavirus,*” “*mouse hepatitis virus,*” “*transmissible gastroenteritis virus,*” “*rotavirus,*” and “*cat*” (Table 1).

#### 2000-2009

A total of 4810 documents was produced in coronavirus literature between 2000 and 2009, and 82.682% of all items were original articles. The United States, China, Canada, Germany, and Netherlands were the most productive countries (*N* = 1679, 1202, 324, 322, and 278 papers, relatively). University of Hong Kong (China), Chinese Academy of Sciences (China), and Chinese University of Hong Kong (China) were the most contributor institutions in this period (5.925, 4.595, and 3.576%, respectively). Yuen form the University of Hong Kong (China) was the most prolific author with 110 papers (2.287%) and the most productive source titles were detected to be *Journal of Virology*, *Virology* and *Advances in Experimental Medicine and Biology* (10, 3.493 and 3.222%, respectively). H-index for this decade was 165 and total number of citations was 189,424 (135,829 without self-citations). Average citations per item were 39.38. The most cited article in this period was an original article written by SARS Working Group, Ksiazek et al., titled “*A novel coronavirus associated with severe acute respiratory syndrome*” published in *The New England Journal of Medicine* in 2003 (Table 1). The most indexed keywords were detected to be “*coronavirus,*” “*SARS-CoV,*” and “*spike protein*” (Table 1). Scientometric network map of the most used keywords showed a starburst pattern in which the keywords of “*coronavirus,*” “*SARS,*” “*SARS coronavirus,*” and “*SARS-CoV*” centered.

#### 2010-2019

The decade of 2010s was the most active period of the literature with 6601 documents. Most documents (*N* = 4146; 62.8%) were open access and 80.7% of all items were original articles. The United States, producing 33.586% of all coronavirus literature, ranked first as ever, followed by China, Germany, Saudi Arabia, and The United Kingdom (22.406, 6.575, 6.454, and 6.166%, respectively). University of Hong Kong (China), National Institutes of Health (USA), Chinese Academy of Sciences (China), and University of California System (USA) stood out from the rest (3.681, 2.787, 2.575, and 2.439%, respectively) and Drosten from Charité – Universitätsmedizin (Germany) was the most prolific author of this period with 113 articles. A total of 6111 articles were analyzed for the citation analysis. H-index of this period was measured as 106. Indexed documents were cited 109,418 times (53,521 times without self-citations). An original article titled “*Isolation of a Novel Coronavirus from a Man with Pneumonia in Saudi Arabia*” written by Zaki indexed in *The New England Journal of Medicine* in 2012 (Table 1). The most indexed keywords were “*coronavirus,*” “*MERS-CoV,*” “*epidemiology,*” and “*SARS-CoV*” (Table 1). Scientometric network analysis of keywords revealed relative keywords such as “*Saudi Arabia,*” “*MERS-CoV,*” “*outbreak,*” *vaccine,*” “*camel,*” and “*zoonosis.*”

### Update for 2020 and COVID-19 (SARS-CoV-2)

By the date of the writing of this study (May 15, 2020), 6679 documents were indexed relevant to CoVid-19 in PubMed and 4605 items were included in WoS databases. All included documents were published and indexed in 2020. Total number of open-access coronavirus articles produced in 2020 was 4605. China ranked first with 1184 documents (25.711%) followed by the United States, the United Kingdom, Italy, Germany, India, Canada, Australia, France, Iran, Switzerland, Brazil, and South Korea (23.822, 9.511, 9.186, 4.039, 3.757, 3.735, 3.648, 3.409, 3.062, 2.953, 2.323, and 2.237%, respectively). The most contributor institutions were University of London (UK), Huazhong University of Science Technology (China), Harvard University (USA), University of California System (USA), Wuhan University (China), University of Hong Kong (China), Chinese Academy of Sciences (China), Zhejiang University (China), University of Oxford (UK) and Fudan University (China) (3.149, 2.758, 2.193, 2.172, 2.041, 1.781, 1.694, 1.607, 1.433, and 1.39%, respectively). Major source titles for 2020 were *Lancet*, *British Medical Journal*, *Journal of Medical Virology*, *Nature*, *Science,* and *Cureus* (*N* = 135, 119, 108, 95, 89, and 78 items, respectively).

## DISCUSSION

It is important to analyze the coronavirus literature based on periods, because it helps us understand the progression of the disease. In the 1980s, the United States and developed countries from Europe were major source countries. During this period, the virus was identified only as an animal disease in the literature, and its biological and genetic structure was investigated. In the second decade we investigated (1990s), the United States and European countries stood out again and the coronaviruses started to be examined in terms of whether it affected the health of animals and people with asthma (Table 1). In fact, in this period, the effects of these viruses on the human respiratory system were underlined in the literature and perhaps early measures could be taken for a possible global eradication of coronaviruses. Of course, this is just our hypothetical and speculative interpretation.

The 2000s can be called as the SARS period in the coronavirus literature. In November 2002, a novel respiratory system disease coined as SARS was identified in China caused by SARS-CoV. This disease caused global anxiety, because it progressed through an outbreak and epidemic in 26 countries and more than 8000 people were affected.^7^ In the literature of the 2000s, China became a major contributor of coronavirus literature because the SARS outbreak originated from Guangdong province of southern China. Almost all most-cited publications in this period are related to SARS and ACE2 protein. SARS-CoV is “*thought to be an animal virus from an as-yet-uncertain animal reservoir, perhaps bats, that spread to other animals (civet cats)*” according to WHO. We want to draw attention to one of the most cited articles in this period titled “*Bats are natural reservoirs of SARS-like coronaviruses*” written by Li et al. published in *Science* in 2005 (Table 1). The authors reported that species of bats were a natural host of coronaviruses closely related to those responsible for the SARS outbreak. In the conclusion paragraph of this manuscript, Li et al. suggested in-depth investigation of reservoir host distribution, animal-animal and human-animal interaction (particularly within the wet-market system), and analyzing genetic diversity of bat-borne viruses to avoid future outbreaks.^8^

In April 2012, a novel lethal zoonotic pathogen, MERS-CoV, was identified in humans in Saudi Arabia and Jordan. A total of 2499 confirmed cases and 858 deaths (mortality rate, 34.3%) were reported from 27 countries. Outbreaks caused by human-human transition in Saudi Arabia in 2014 and South Korea in 2015 occurred.^9^ In 2010s, Saudi Arabia became one of the major sources in coronavirus literature. Almost all most-cited articles were related to MERS in this period.

We found only one scientometric document relevant to coronavirus literature. It was a letter with limited data and no tables or images revealing scientometric networks. The authors reported that the United States and China had primary roles in the literature.^10^

## CONCLUSIONS

In late 2019, a novel coronavirus causing SARS-like pneumonia was identified. This novel virus was temporarily named as SARS-CoV-2 and the disease has been coined as COVID-19 by the WHO.^2^ By the date of writing this study, a total of 167,682 confirmed patients and 6456 deaths were reported from 157 countries and territories, and the COVID-19 outbreak has been officially declared as pandemic. We hypothesized that the COVID-19 pandemic could be prevented if the suggestions of the articles previously published, such as that by Lie et al., were taken into consideration and the wet-markets were completely closed to cut off the human-animal interaction. Scientometric studies should be performed in certain diseases that previously caused epidemics to prevent global spread in the future and to take urgent measures.
